# Loss of DIAPH3 accelerates glioma genesis in mice

**DOI:** 10.1038/s41419-026-08652-x

**Published:** 2026-03-23

**Authors:** Georges Chehade, Irene Durá, Nuria Ruiz-Reig, Devid Damiani, Eva On-Chai Lau, Julie Lelotte, Nicolas Joudiou, Mohamed Aittaleb, Fadel Tissir

**Affiliations:** 1 Developmental Neurobiology Laboratory, Institute of Neuroscience, Université catholique de Louvain, Brussels, Belgium; 2 Department of Neuropathology, Saint-Luc University Hospital, Brussels, Belgium; 3 Nuclear and Electron Spin Technologies Platform, Louvain Drug Research Institute, Université catholique de Louvain, Brussels, Belgium; 4 College of Health and Life Sciences, Hamad Bin Khalifa University, Doha, Qatar; 5 Present Address: Department of Neurobiology and Molecular Medicine, IRCCS Fondazione Stella Maris, Pisa, Italy

**Keywords:** Cancer genomics, Oncogenesis

## Abstract

DIAPH3 is a master regulator of the cytoskeleton with key roles in cell division. In the mouse brain, DIAPH3-deficient neural stem cells exhibit abnormalities in karyokinesis and cytokinesis, leading to cell cycle arrest, aneuploidy, and mitotic catastrophe. Here, we investigated the role of DIAPH3 in glioma genesis in mouse models. We selectively deleted the *Diaph3* and *Trp53* genes in the mouse cerebral cortex and thoroughly analyzed single (*Diaph3* cKO and *Trp53* cKO) and double (dcKO) conditional knockout mice. The tumors appeared earlier in dcKO than in *Trp53* cKO mice, and this was associated with increased whole chromosome copy number alterations, endogenous DNA damage, and shorter survival of dcKO mice. We performed a comparative transcriptomic analysis prior to the onset of tumors and identified changes in cancer gene signatures specifically in dcKO, suggesting that the loss of DIAPH3 hastens the tumorigenic process. We isolated cancer stem-like cells and assessed their sensitivity to ionizing radiation and found that DIAPH3 regulates the resistance of glioma stem-like cells to irradiation. Our data suggest that DIAPH3 has a tumor-suppressor function and that its deficiency promotes aneuploidy and genome instability, accelerating tumorigenesis and leading to early onset of high-grade diffuse glioma with DNA damage, and resistance to ionizing radiation.

## Introduction

Diaphanous-related formin (DIAPH) 3 is a member of the formin protein family, an evolutionarily conserved multidomain protein, which in mammals includes 15 members grouped into seven subfamilies [1]. Formins are primarily known for their cardinal role in actin nucleation, elongation, polymerization, and remodeling of actin filaments [2]. Beyond their interaction with actin, some formins also associate with microtubules and regulate their dynamics, thereby contributing to the structural organization of the cytoskeleton [3]. Hence, formins regulate critical cellular functions such as division, adhesion, motility, and intracellular transport.

The diaphanous-related formin subfamily comprises 3 members with different expression profiles and distinct functions [4–6]. DIAPH3 plays a significant role in cell division, especially during cytokinesis [4, 7]. It also regulates karyokinesis by organizing the mitotic spindle [8] and the spindle assembly checkpoint [9]. Consistent with these pivotal functions in mitosis, DIAPH3 is highly expressed in neural progenitor cells in the developing mouse brain, and its loss has been linked to impaired cell division, chromosomal instability (CIN), aneuploidy, depletion of neural progenitor cells, and abnormal cortical histogenesis [8, 9].

Errors during cell division often lead to mitotic catastrophe, which triggers cell death or senescence, hindering cell proliferation. Nevertheless, aneuploidy -a state of abnormal chromosome number- is a hallmark of highly proliferative cancer cells. The outcome of chromosome missegregation can vary from promoting to inhibiting tumorigenesis according to the severity of chromosomal instability (CIN) and tissue context [10]. Moreover, aneuploidy is often associated with poor prognosis, progression, metastasis, and resistance to therapeutic treatment of malignant tumors [11]. Glioblastoma (GBM), the most common and aggressive primary brain tumor in adults, is characterized by a high degree of aneuploidy [12]. This implies that aneuploidy is an important driver of disease onset, progression, and/or recurrence. For instance, chromosome 7 gain and chromosome 10 loss are frequent early events in GBM [13] and contribute to increased expression of the oncogene *EGFR* and reduced expression of the tumor suppressor *PTEN*, respectively. Additionally, focal loss of locus 13q22.1, where DIAPH3 is encoded [14], is also frequently observed in GBM, suggesting that this region could be implicated in GBM physiopathology.

Reasoning that the loss of DIAPH3 contributes to aneuploidy, that DIAPH3 deletions are commonly found in GBM, and that its expression is associated with a better survival of GBM patients [15], we investigated the function of DIAPH3 in the genesis and progression of glioma in a mouse model lacking Trp53 in neural progenitors and their progeny. Trp53 deficiency prevents cells, which accumulate mutations and/or DNA damage, from activating the DNA damage response or undergoing apoptosis, thereby enabling tumorigenesis. We report that cortex-specific deletion of *Diaph3* on *Trp53* background fosters early onset development of high-grade diffuse glioma and increases the level of aneuploidy, endogenous DNA damage and resistance to ionizing radiation.

## Materials and methods

### Mice

Animals were housed under a standard 12-h dark/12-h light cycle. The temperature was between 20 and 24 °C, and the humidity was between 40% and 60%. The following mouse lines were used: *Emx1-Cre* [16], *Diaph3*^*F/F*^ [9] and *Trp53*^*F/F*^ [17]. To generate *Emx1-Cre;Diaph3*^*F/F*^ mice (*Diaph3* cKO), we crossed *Emx1-Cre;Diaph3*^*F/F*^ males with *Diaph3*^*F/F*^ females. To produce *Emx1-Cre;Trp53*^*F/F*^ mice (*Trp53* cKO), we crossed *Emx1-Cre;Trp53*^*F/F*^ males with *Trp53*^*F/F*^ females. To generate *Emx1-Cre;Diaph3*^*F/F*^*;Trp53*^*F/F*^ mice (dcKO), we crossed *Emx1-Cre;Diaph3*^*F/F*^*;Trp53*^*F/F*^ males with *Diaph3*^*F/F*^*;Trp53*^*F/F*^ females. The mice were inspected daily by the animal caretaker. In case of morphological or behavioral changes (e.g., hunched back, decreased water and food intake or lethargy), mice were euthanized, and the brain tumors were harvested and processed as described below. All studies were conducted in a mix of males and females. The age of animals is specified in the results section for each experiment.

### Brain tumor tissue preparation and sectioning

For hematoxylin and eosin staining and immunohistofluorescence, mice were transcardially perfused with 4% paraformaldehyde (Merck, 1.04005.1000). Brain tumors were harvested and postfixed in 4% paraformaldehyde overnight at 4 °C, washed in PBS and cryoprotected by gradients of sucrose solution. The brains were embedded in optimal cutting temperature compound (Thermo Fisher Scientific, 23730625), sectioned into 20-µm slices with a Leica CM1900 UV cryostat (Leica, Germany) and then mounted on positively charged slides (Thermo Fisher Scientific, 10149870).

### Ultra-high-field 11.7 Tesla magnetic resonance imaging

MRI was performed using an 11.7 Tesla Bruker Biospec MRI system (Bruker, Germany) equipped with a 1H quadrature birdcage coil (21 mm inner diameter) (RAPID Biomedical, Germany). Mice were anesthetized using isoflurane mixed with air. Respiration was continuously monitored, while the animal’s temperature was maintained with a warm blanket. Brain tumors were detected using a rapid acquisition with relaxation enhancement (RARE) sequence (TR = 2500 ms; effective echo time (TEeff) = 30 ms; RARE factor = 8; FOV = 2 × 2 cm; matrix 200 × 200; 26 contiguous slices of 0.4 mm, NA = 10). The brain tumor volume was calculated by multiplying the thickness of a slice (0.4 mm) by the sum of the tumor areas obtained using Fiji software (ImageJ).

### Glioma stem-like cell culture and irradiation

Glioma stem-like cell isolation and culture protocols were adapted from Seidel and colleagues [18]. Mouse brain tumors were dissociated using 20 U/mL papain (Merck, P4762) and 50 U/mL DNase I (Merck, 11284932001) for 30 min at 37 °C. The cells were cultured in medium containing DMEM/F-12 and GlutaMAX™ (Thermo Fisher Scientific, 31331028) supplemented with penicillin/streptomycin/amphotericin B (Merck, A5955), 5 mM HEPES buffer (Thermo Fisher Scientific, 15630106), B-27™ supplement, minus vitamin A (Thermo Fisher Scientific, 12587010), 20 ng/mL recombinant mouse EGF (Bio-Techne, 2028-EG) and 20 ng/mL recombinant mouse FGF basic (Bio-Techne, 3139-FB). Glioma stem-like cells (before passage 10) were irradiated (5 Gy, equivalent to 141 s) at room temperature using an IBL 637 (^l37^Cs) gamma irradiator (Cisbio International, France). Thereafter, extreme limiting dilution analysis was performed as described previously [19]. In brief, irradiated and nonirradiated cells were cultured in 96-well plates at different concentrations (40, 20, 10 and 5 cells/well; 12 replicates/concentration). Fourteen days later, the wells with positive cultures (at least one 100-µm spheroid) were counted, and the active cell frequency was calculated using the online tool https://bioinf.wehi.edu.au/software/elda/. Images were obtained with a Zeiss Axio Vert.A1 microscope equipped with a Zeiss Axiocam 503 mono camera (Zeiss, Germany).

## Results

### DIAPH3 loss hastens high-grade diffuse glioma genesis in Trp53 cKO mice

**Fig. 1:**
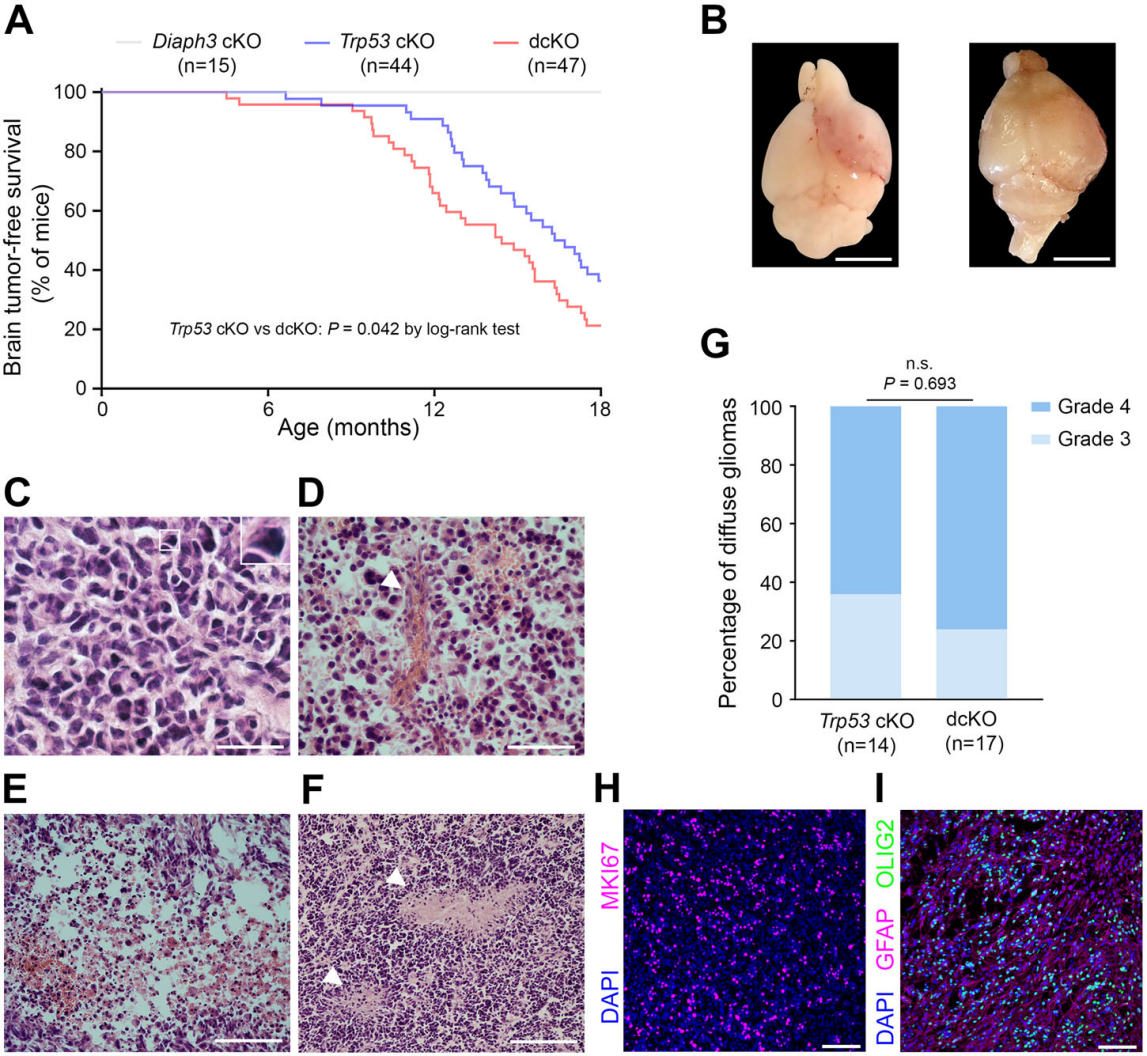
DIAPH3 loss causes early glioma-related death in *Trp53*-deficient mice. **A** Mouse survival estimated by Kaplan‒Meier analysis (*Trp53* cKO: median survival = 16.3 months, 95% CI: 14.5–18.2, *n* = 44; dcKO: median survival = 14.4 months, 95% CI: 11.6–17.3, *n* = 47; *P* = 0.042 by log-rank test). **B** Illustrative images of whole brains showing tumors. Hematoxylin and eosin staining of brain tumor sections showing astroglial morphology, nuclear atypia and hypercellularity (**C**), microvascular proliferation (**D**, arrowhead), necrosis (**E**) and pseudo-palisading necrosis (**F**, arrowheads). The inset in (**C**) is a zoom on a nuclear atypia. **G** Histology-based grading of end-stage diffuse gliomas (*Trp53* cKO: grade 3 = 36%, grade 4 = 64%, *n* = 14; dcKO: grade 3 = 24%, grade 4 = 76%, *n* = 17; *P* = 0.693 by Fisher’s exact test). **H, I** Glioma sections stained with MKI67 (**H**, magenta), GFAP (**I**, magenta) and OLIG2 (**I**, green) antibodies. Nuclei were counterstained with DAPI (blue). Scale bars: **B** 5 mm; **C** 20 µm; **D** 50 µm; **E**, **F**, **H**, **I** 100 µm.

**Fig. 2:**
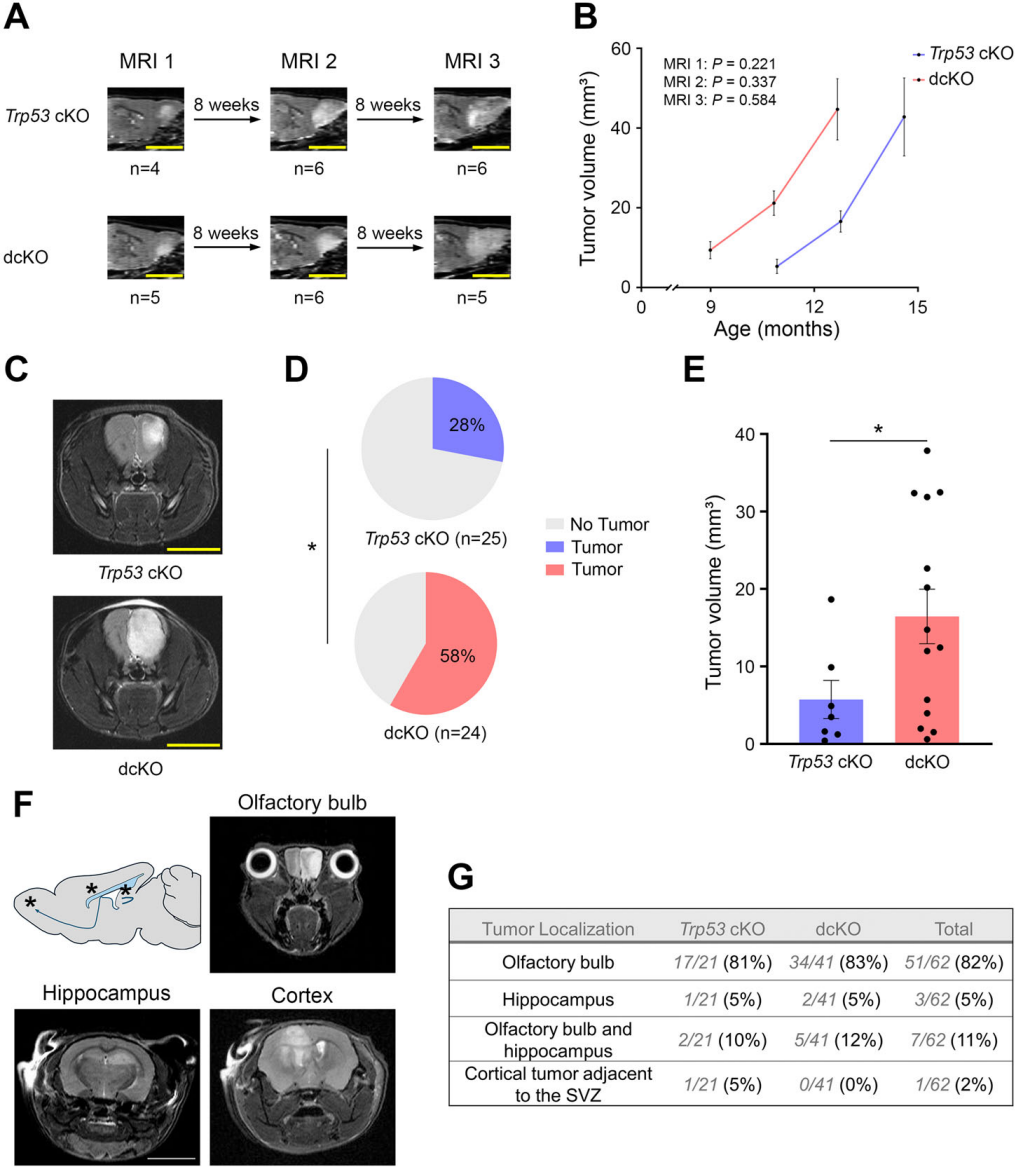
DIAPH3 deficiency accelerates high-grade diffuse glioma development. **A** Schematic diagram illustrating MRI monitoring to evaluate the growth of brain tumors. **B** Tumor volumes measured on MRI 1 scans (*Trp53* cKO: mean = 5.297 mm³, SEM = 1.782, *n* = 4; dcKO: mean = 9.398 mm³, SEM = 2.156, *n* = 5; *P* = 0.221 by Mann–Whitney test), MRI 2 scans (*Trp53* cKO: mean = 16.582 mm³, SEM = 2.664, *n* = 6; dcKO: mean = 21.181 mm³, SEM = 3.067, *n* = 6; *P* = 0.337 by Mann–Whitney test) and MRI 3 scans (*Trp53* cKO: mean = 42.785 mm³, SEM = 9.775, *n* = 6; dcKO: mean = 44.691 mm³, SEM = 7.684, *n* = 5; *P* = 0.584 by Mann–Whitney test). **C** Illustrative MRI scans from *Trp53* cKO and dcKO showing brain tumors in hypersignal. **D** Brain tumor frequency on MRI scans between 9 and 11 months (*Trp53* cKO: frequency = 28%, *n* = 25; dcKO: frequency = 58%, *n* = 24; *P* = 0.032 by Pearson’s chi-squared test). **E** Brain tumor volumes measured on MRI scans between 9 and 11 months (*Trp53* cKO: mean = 5.735 mm³, SEM = 2.467, *n* = 7; dcKO: mean = 16.454 mm³, SEM = 3.518, *n* = 14; *P* = 0.022 by independent-samples *t* test). Scale bars: (**A**, **C**) 5 mm. **F** Localization of glioma in *Trp53* cKO and dcKO mice studied by MRI. The tumors developed in the olfactory bulbs, hippocampus, and subventricular zone. **G** Distribution of the tumors according to the localization and genotype.

To test the potential relationship between loss of DIAPH3, aneuploidy, and tumorigenesis, we generated cortex-specific conditional knockout mice for *Diaph3* (*Diaph3* cKO), *Trp53* (*Trp53* cKO) and *Diaph3* and *Trp53* (dcKO) by crossing *Emx1-Cre* mice with *Diaph3*^*F/F*^ mice, *Trp53*^*F/F*^ mice, and *Diaph3*^*F/F*^; *Trp53*^*F/F*^ mice, respectively (Supplementary Fig. S1A, B). While *Diaph3* cKO mice survived healthily up to 24 months and didn’t develop tumors, some *Trp53* cKO and dcKO mice developed morphological and behavioral phenotypes (e.g., hunched back, decreased water and food intake, or lethargy) and were euthanized starting from the ages of 6.5 months and 4.5 months, respectively (Fig. 1A). Necropsy of affected mice revealed brain tumors (Fig. 1B), whereas no tumors were detected in single *Diaph3* cKO mice. Accordingly, lifespan was reduced, with a median survival of 16.3 months (95% CI: 14.5–18.2) for *Trp53* cKO mice and 14.4 months (95% CI: 11.6–17.3) for dcKO mice (*P* = 0.042 by log-rank test; Fig. 1A). Quantitative reverse transcription PCR analysis confirmed the loss of *Diaph3* in dcKO brain tumors (Supplementary Fig. S1C, D). In addition to being expressed in *Trp53* cKO gliomas, *Diaph3* mRNA was exclusively expressed in neural stem cells and their early progeny in the non-tumoral adult mouse brain (Supplementary Fig. S1E). Histological analysis of the brain tumors showed features of high-grade diffuse gliomas, including astroglial morphology, nuclear atypia, and hypercellularity (Fig. 1C). The distinction between grade 3 and grade 4 tumors was based on the presence of microvascular proliferation (Fig. 1D) and necrosis (Fig. 1E, F). Overall, approximately 70% of the end-stage tumors were grade 4 diffuse gliomas, with no difference between *Trp53* cKO and dcKO mice (*P* = 0.693; Fig. 1G). The tumors were highly proliferative (Fig. 1H) and immunoreactive for GFAP and OLIG2 (Fig. 1I), corroborating the histological diagnosis. By the age of 18 months, 64% of *Trp53* cKO mice died of high-grade diffuse gliomas compared to 79% of dcKO mice (Fig. 1A). The difference in survival between *Trp53* cKO and dcKO mice could be related to earlier tumor development or faster tumor growth in dcKO mice. To discriminate between the two possibilities, we performed a time-course analysis using an ultra-high-field 11.7 Tesla MRI scanner. We examined animals every eight weeks and detected earlier tumor development in dcKO mice, but no difference in tumor growth rate was observed between *Trp53* cKO and dcKO mice (Fig. 2A, B). This was confirmed by MRI performed at the age of 9–11 months, which showed brain tumors in only 28% of *Trp53* cKO mice *versus* 58% of dcKO mice (*P* = 0.032; Fig. 2C, D). dcKO mice had larger tumors than *Trp53* cKO mice (*P* = 0.022; Fig. 2C, E). We also used MRI scans to analyze the localization of tumors in 62 affected mice (21 *Trp53* cKO and 41 dcKO; Supplementary Table S1). We found that 82% (51/62) of tumorigenic mice developed glioma in olfactory bulbs (OB), 11% (7/62) in OB and hippocampus, 5% in hippocampus (3/62), and 2% (1/62) in the vicinity of the subventricular zone extending dorsally to the cortex and ventrally to the striatum (Fig. 2F, G).

Overall, these results show that the loss of DIAPH3 promotes early-onset high-grade diffuse gliomas in *Trp53-*deficient mice.

### DIAPH3 loss alters the transcriptional landscape accelerating the tumorigenic process

**Fig. 3:**
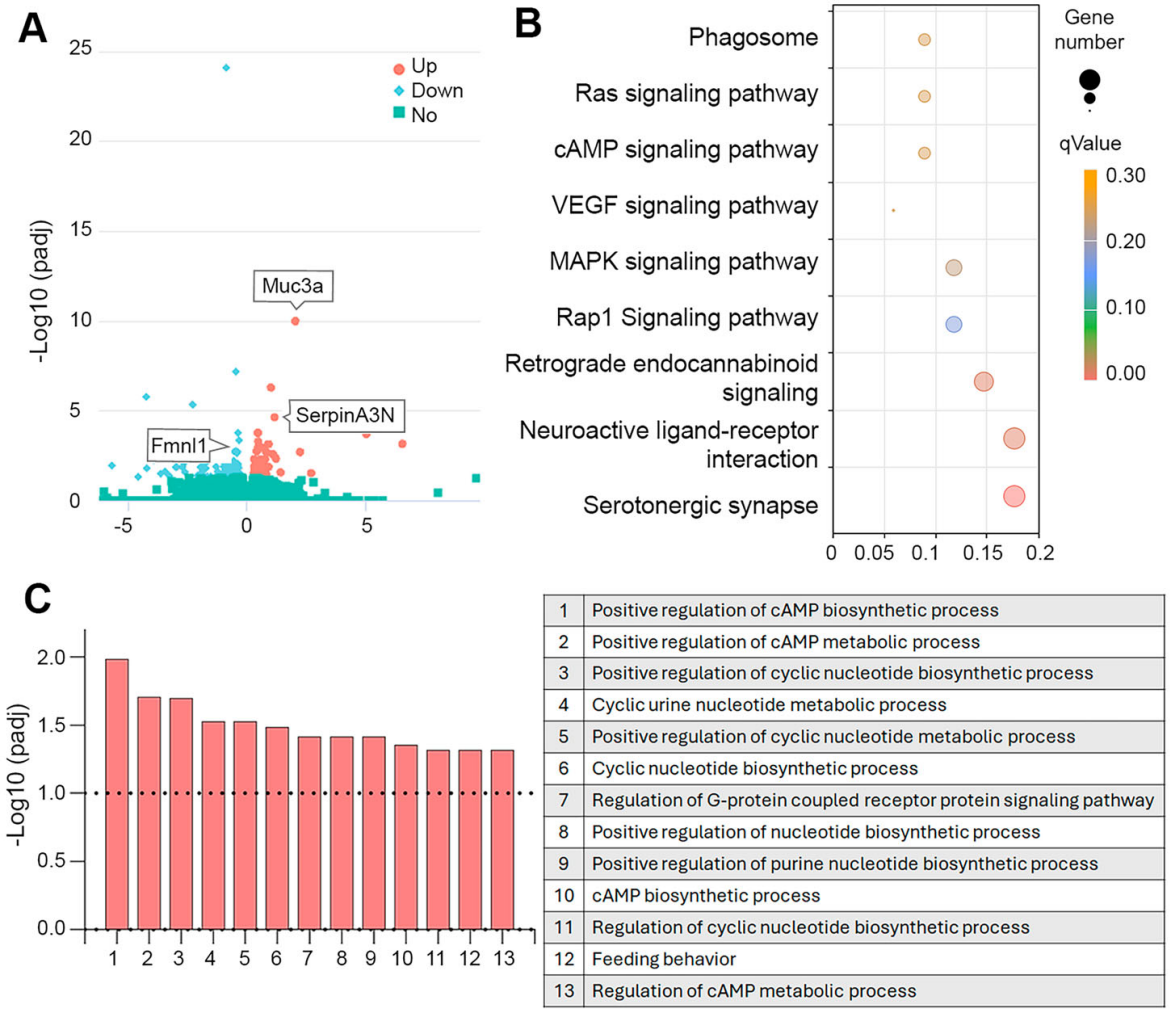
DIAPH3 deficiency promotes early tumor initiation. RNA-seq analysis results from 4-months-old OB (*n* = 4 per group) identified a total of 126 significantly differentially expressed genes (absolute log2FoldChange > 0, padj < 0.05). **A** Volcano plot illustrates 40 upregulated and 86 downregulated genes in the dcKO group versus the Trp53 cKO (log2FoldChange > 0, padj < 0.05). **B** KEGG analysis results depicting changes in three metabolic pathways (Gene Number ≥ 5, qValue < 0.05). Low qValues are shown in red while high qValues are in orange. The size of the circle is proportional to the number of enriched genes. **C** Results from GO analysis (right panel) identified statistically significant alterations in 13 different functions (right panel) in dcKO compared to Trp53 cKO (padj < 0.05). Abbreviations: padj, *p*-value adjusted; GO Gene Ontology, KEGG Kyoto Encyclopedia of Genes and Genomes.

To gain insight into the molecular changes underlying the earlier initiation of glioma in dcKO mice, we performed a comparative transcriptomic analysis of OB from 4-months-old Trp53 cKO and dcKO mice, prior to tumor development using RNA sequencing. A total of 126 genes were differentially expressed (DEGs) in the dcKO as compared to Trp53 cKO. Forty were upregulated, whereas 86 were downregulated (Fig. 3A and Supplementary Table S2). Sixty DEGs (±48%) have been previously linked to different types of cancer (Supplementary Table 3). These include for instance SerpinA3N, Tox4, Tpbg and Muc3a. Recent studies have associated SERPIN3A, the human orthologue of SerpinA3n, with several tumorigenic processes including cell proliferation, invasion, migration, and epithelial to mesenchymal transition [20]. TOX4 has been identified as part of a prognostic gene signature for GBM [21]. TPBG is overexpressed in numerous cancers including colorectal, ovarian, and gastric cancers where it is believed to alter cell adhesion dynamics and promote cell migration and invasion, and epithelial-mesenchymal transition [22]. MUC3A dysfunction is associated with various cancers such as clear cell renal cell carcinoma, colorectal cancer, and non-small cell lung cancer [23, 24]. It has been suggested to promote tumor proliferation, invasion and chemoresistance. Finally, the expression Fmnl1, another formin protein that regulates actin dynamics in mitotic cells, was altered. Human FMNL1 is implicated in various cancers, potentially acting as a pro-metastatic oncogene. It’s believed to promote tumor progression and metastasis by influencing cell migration and potentially mediating the epithelial-mesenchymal transition, particularly in clear cell renal cell carcinoma [25].

KEGG pathway enrichment analysis emphasized several oncogenic pathways such VEGF, MAPK, cAMP, RAS, MAPK, cAMP and Rap1 (Fig. 3B). VEGF plays a crucial role in cancer development and progression, partially by promoting the formation of new blood vessels, which tumors need to grow and expand [26]. The MAPK signaling influences cell growth, division, differentiation and survival. In cancer, dysregulation of this pathway can lead to uncontrolled cell proliferation, increased survival, and resistance to therapies [27]. RAS and Rap1 are small GTPases that act as molecular switches, influencing cell adhesion, migration, and survival, which are crucial processes for tumor development [28]. cAMP is a second messenger with complex and context-dependent roles in cancer. cAMP can either promote or inhibit cell growth and survival according to the type of cancer and the surrounding microenvironment [29]. Other pathways downregulated in dcKO comprise serotonergic synapses and retrograde endocannabinoid signaling. This alteration may reflect a loss of neurons or change in the microenvironment which could favor tumorigenesis by hindering synaptic transmission. GO enrichment analysis confirmed alterations in two intertwined pathways, namely G-protein coupled receptor signaling and cyclic nucleotide metabolic process (especially cAMP) (Fig. 3C). GPCRs activate G-proteins (e.g., Ras and Rap1) and trigger downstream signaling cascades including cAMP production via adenylyl cyclase. Some GPCRs drive oncogenic signaling and activate both pro-survival and anti-apoptotic pathways [30]. These alterations in gene expression in dcKO mice but not in Trp53 cKO prior to tumor detection support the hypothesis of an earlier onset of tumor initiation in this model. In addition to DEGs involved in tumorigenesis, 10 DEGs were related to the immune system, 5 to angiogenesis, and 3 to the extracellular matrix and tumor environment (Supplementary Table 3).

### DIAPH3 loss increases whole chromosome copy number alterations, DNA damage, and resistance to radiation

**Fig. 4:**
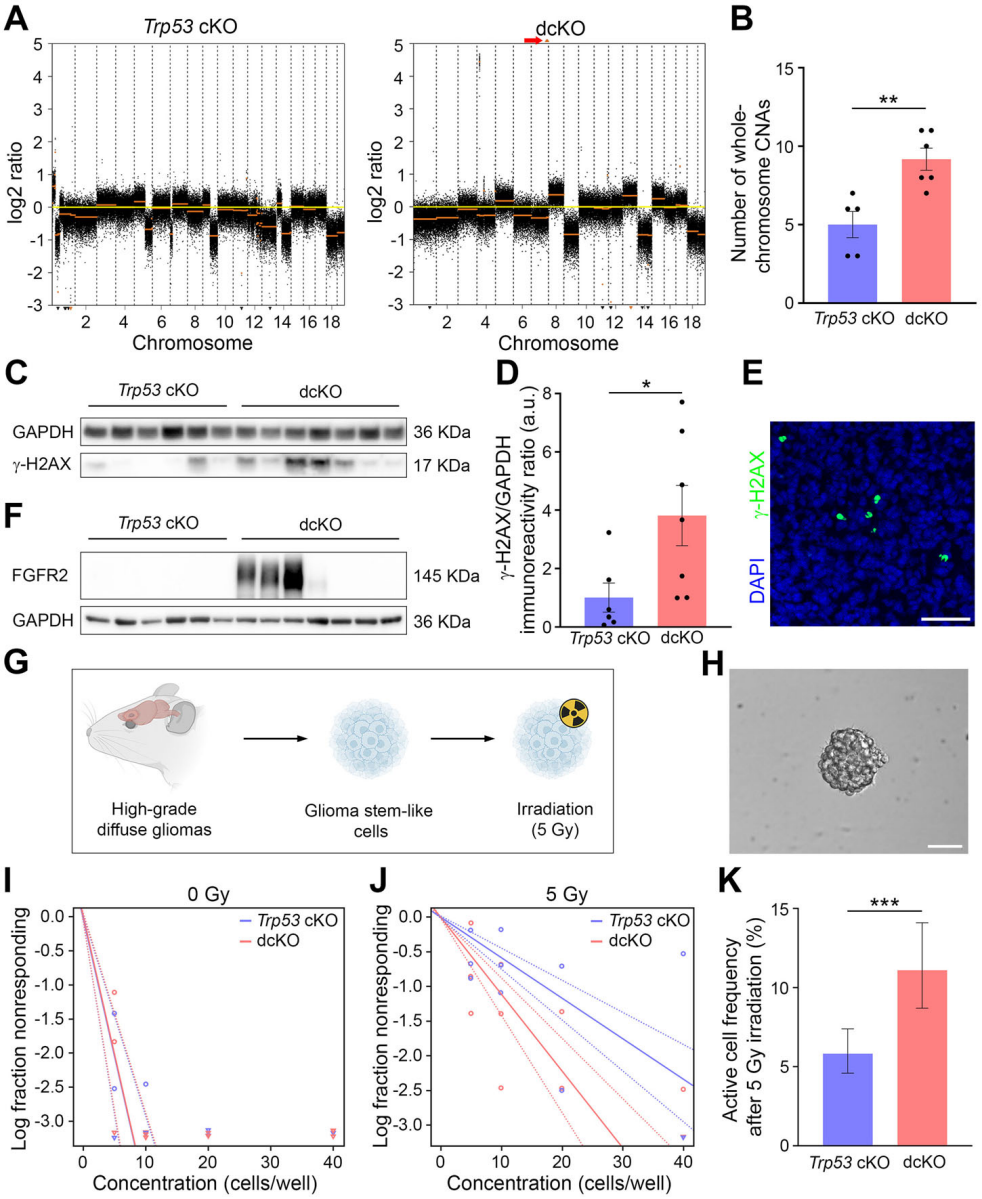
DIAPH3 deficiency triggers aneuploidy, endogenous DNA damage and resistance to irradiation. **A** Representative copy number alteration (CNA) plots for *Trp53* cKO (left) and dcKO (right) gliomas. The red arrow points to the high-level amplification of *Fgfr2* in dcKO. **B** Number of whole-chromosome CNAs in *Trp53* cKO and dcKO gliomas (*Trp53* cKO: mean = 5.000 chromosomes, SEM = 0.837, *n* = 5; dcKO: mean = 9.167 chromosomes, SEM = 0.703, *n* = 6; *P* = 0.004 by independent-samples *t* test). Western blot analysis (**C**) and relative quantification (**D**) of γ-H2AX in glioma extracts (*Trp53* cKO: mean = 1.002 a.u., SEM = 0.500, *n* = 6; dcKO: mean = 3.813 a.u., SEM = 1.034, *n* = 7; *P* = 0.022 by Mann–Whitney test). **E** Glioma sections stained with γ-H2AX (green) antibody. Nuclei were counterstained with DAPI (blue). **F** Western blot analysis of FGFR2 in high-grade diffuse glioma extracts (*Trp53* cKO: *n* = 6; dcKO: *n* = 7). **G** Schematic diagram illustrating the isolation of glioma stem-like cells from high-grade diffuse gliomas, followed by irradiation. **H** Illustrative brightfield microscope image of a glioma stem-like cell spheroid. **I**, **J** Extreme limiting dilution analysis (ELDA) plots in *Trp53* cKO and dcKO glioma stem-like cells before (I; *n* = 4 for each genotype; *P* = 0.947) and after (J; *n* = 4 for each genotype; *P* < 0.001) 5 Gy irradiation. The log-active cell fraction is indicated by the slope of the line, and the 95% CI by the two dotted lines. The arrowhead indicates a full response (i.e., spheroids observed in all the tested wells) at the corresponding cell concentration. **K** Active cell frequency in glioma-stem like cell cultures after 5 Gy irradiation (*Trp53* cKO: frequency = 5.82%, 95% CI: 4.59–7.39, *n* = 3; dcKO: frequency = 11.10%, 95% CI: 8.70–14.10, *n* = 3; *P* < 0.001 by ELDA). Scale bars: (**E**, **H**) 50 µm. CNA copy number alteration.

DIAPH3 deficiency undermines the spindle assembly checkpoint and disrupts mitotic division, leading to CIN and increased aneuploidy in embryonic neural stem cells [8, 9]. To test the relationship between DIAPH3, aneuploidy and glioma development, we conducted shallow whole-genome sequencing on DNA from *Trp53* cKO and dcKO tumours. Copy number alteration (CNA) plots (Fig. 4A and Supplementary Fig. S2A, B) showed a higher number of whole-chromosome CNAs in dcKO mice than in *Trp53* cKO mice (*P* = 0.004; Fig. 4B), whereas the number of focal CNAs (CNAs of limited size frequently enriched for cancer driver genes) was not grossly affected (*P* = 0.435; Supplementary Fig. S2C). These results strongly suggest that DIAPH3 loss specifically favors whole-chromosome CNAs (i.e., aneuploidy) in high-grade diffuse gliomas. Notably, some of the whole-chromosome CNAs were recurrent in tumors, regardless of DIAPH3 status (i.e., loss of chromosomes 9, 12, 18, and 19; Supplementary Fig. S2D), suggesting that these chromosomes may harbor relevant genes for glioma genesis, such as *Pten* (a key tumor-suppressor gene in GBM, located on chromosome 19).

Aneuploidy is believed to increase DNA damage through oxidative stress (increase in reactive oxygen species) [31] and replication stress (stalled replication forks) [32]. In line with these observations, dcKO diffuse gliomas showed higher levels of γ-H2AX than *Trp53* cKO diffuse gliomas (*P* = 0.022; Fig. 4C, D and Supplementary File 1A). γ-H2AX immunohistostaining showed a pan-nuclear signal (Fig. 4E). Despite the presumed increase in DNA damage, the tumor growth rates were similar between the two genotypes (Fig. 2A, B), suggesting that dcKO tumors developed a tolerance to these alterations. In search of the underlying mechanisms, we conducted transcriptomic analysis of *Trp53* cKO and dcKO diffuse gliomas (Supplementary Fig. S3A). We identified 417 differentially expressed genes, with 89 upregulated and 328 downregulated genes in the dcKO tumors (Supplementary Fig. S3B). Among the top differentially expressed genes, *Fgfr2* was highly upregulated in dcKO tumors (log2Fold Change) = 7.23, *Padj* = 8.10e-21; Supplementary Table S4). This increase, confirmed at the protein level (in four out of the seven tested dcKO tumors, Fig. 4F and Supplementary File 1B), was associated with amplification of the *Fgfr2* gene (red arrow; Fig. 4A ). Importantly, FGFR2 plays a role in DNA damage repair in GBM cells, and gain-of-function mutations in *FGFR2* correlate with activation of the DNA damage pathway [33]. Hence, the increase in FGFR2 levels may be involved in the repair of DNA damage in dcKO diffuse gliomas and constitute a mechanism of tolerance to aneuploidy.

Because a previous study implicated the FGFR2-mediated DNA damage repair in resistance to radiotherapy in GBM [33], we hypothesized that this mechanism of DNA damage tolerance can affect the response of glioma stem cells (GSC), which play an essential role in GBM recurrence [34], to ionizing radiation. To test this hypothesis, we compared the response of GSC derived from high-grade diffuse gliomas to ionizing radiation (Fig. 4G, H). Extreme limiting dilution analysis underscored a similar baseline active cell frequency between *Trp53* cKO and dcKO mice (*P* = 0.947; Fig. 3I). However, after 5 Gy irradiation, there was a more pronounced reduction in active cell frequency in *Trp53* cKO than in dcKO (*P* < 0.001; Fig. 4J, K), suggesting a higher resistance to ionizing radiation in dcKO tumors.

Altogether, our results show that DIAPH3 loss triggers whole-chromosome CNAs and endogenous DNA damage and is associated with increased resistance to ionizing radiation.

## Discussion

Consistent with previous reports linking CIN to tumor formation [10], our study shows that DIAPH3 loss accelerates the onset of glioma genesis in *Trp53*-deficient mice, strongly supporting a tumor-suppressor role for DIAPH3. In the adult brain, *Diaph3* is specifically expressed in neural stem cells, to which growing evidence points as the origin of mouse gliomas and human GBM [35]. Through defective nuclear division, DIAPH3 loss increases CIN in these cells, promoting their neoplastic transformation and genesis of diffuse gliomas with high-level aneuploidy in a *Trp53*-KO background. However, DIAPH3 loss alone is not sufficient to induce tumors, presumably because aneuploid cells are eliminated by *Trp53*-dependent mechanisms [31].

While some publications have previously connected DIAPH3 deficiency to enhanced amoeboid motility in cancer cells via decreased microtubule stability [36, 37], substantial evidence supports a more prominent role of DIAPH3 in cancer invasion and metastasis [38, 39]. Notably, these invasion-related roles do not contradict tumor-repressing activity as they likely operate during distinct stages of tumor development and progression. We propose that the involvement of DIAPH3 in spindle microtubule–kinetochore interactions during mitosis, on the one hand and in actin filopodia/lamellipodia during cell migration, on the other hand, underlie its tumor-suppressor and pro-invasion functions, respectively. This dual role is not unprecedented since other proteins encoded by tumor suppressor genes (e.g., BRCA1) have also been reported to favor metastatic dissemination [40].

The relationship between CIN, aneuploidy, and cancer therapies is not fully understood. While some studies suggest that high levels of CIN enhance resistance to treatment, others imply that CIN sensitizes cancer cells to therapies [15, 41]. Our study suggests that aneuploidy-induced DNA damage promotes the acquisition of tolerance mechanisms (e.g., *Fgfr2* amplification) that confer cancer cells with increased resistance to the DNA-damaging ionizing radiation. This is in contrast with a previous study that showed that ionizing radiation induces chromosome missegregation in U251 cells and that inhibiting CIN increases the resistance to radiation [42]. The duration of exposure of cells to CIN could affect their response to radiation. In our study, dcKO tumors grew in a context of high levels of CIN and aneuploidy and developed tolerance mechanisms and resistance to radiotherapy, whereas in Bakhoum’s study [42], the sensitivity to ionizing radiation was assessed directly after acute manipulation of CIN in the GBM cell line U251.

The intrinsic activation of DNA damage response in GBM contributes to resistance to ionizing radiation, especially in cancer stem-like cells [43]. Importantly, we identified an amplification of *Fgfr2* in *Diaph3*-deficient tumors, which might enhance DNA damage repair. Indeed, Ma and colleagues showed that FGFR2 phosphorylates PTEN on tyrosine 240, promoting RAD51-mediated DNA repair by homologous recombination and enhancing resistance to irradiation in GBM cells [33]. Furthermore, gain-of-function mutations in *FGFR2* are associated with activation of the ATM/CHEK2 DNA damage pathway [44].

Analysis of the TCGA data revealed that 35% of GBM patients have a copy number loss of DIAPH3 that is concomitant with a larger chromosomal deletion involving locus 13q22.1. Moreover, the DIAPH3 expression level is associated with the overall survival of patients with a methylated MGMT promoter. Understanding the regulation of DIAPH3 expression and manipulating its activity are thus essential. *DIAPH3* expression is a tightly regulated process. In embryonic development, *Diaph3* is ubiquitously expressed before the ninth embryonic day, after which its expression becomes tissue-restricted. In the adult mouse brain, *Diaph3* expression is confined to neural stem/progenitor cells and excluded from postmitotic cells. Two different types of modulators of DIAPH3 activity have been identified previously: the small molecule inhibitor, which targets the FH2 domain of formins (SMIFH2) [45], and intramimics 01 and 02 (IMM-01 and IMM-02), which are activators [46]. Notably, in zebrafish models, IMM-01 and IMM-02 showed lower toxicity than SMIFH2 [47]. However, a major limitation of these modulators is their lack of specificity, since they modify the activity of multiple formin family members, boosting the probability of potential side effects. Hence, the search for molecules able to specifically target DIAPH3 remains primordial.

Targeting DIAPH3 in GBM is interesting given its dual role in chromosomal stability and migration of cancer cells. Restoring the function of DIAPH3 might seem attractive to decrease CIN and sensitize tumors to ionizing radiation. However, it could enhance the invasive behavior of the tumor cells. From a clinical perspective, DIAPH3 inhibition might be more suitable, as this would cause acute CIN, reduce proliferation, and enhance cell death, as seen with inhibition of the kinesin KIF2C in GBM stem-like cells [48]. SMIFH2 has been shown to be cytotoxic and to induce defective cytokinesis in a mouse fibroblast line [45]. Particular attention should be given to the toxicity of such inhibitors, especially their hematological toxicity, given the role of DIAPH3 in erythropoiesis [7], and to their capacity to cross the blood‒brain barrier. Gene therapy has also been successfully used to treat certain diseases, including cancer [49]. By modulating its expression, DIAPH3 could serve as a therapeutic target to improve the treatment outcome.

Despite significant advances in the molecular profiling of GBM, its treatment and prognosis have not improved over the last twenty years, with a median OS rarely surpassing 15 months even with optimal therapies involving microsurgical resection followed by radiotherapy combined with concurrent and adjuvant temozolomide treatment [50]. Hence, there are still critical gaps in the understanding of this disease’s pathophysiology. In this study, we identified DIAPH3 as a key regulator of glioma onset and aggressiveness. Mechanistically, we provide evidence that DIAPH3 deficiency promotes aneuploidy, thereby accelerating the tumorigenic process and emergence of diffuse gliomas with higher levels of endogenous DNA damage and radioresistance. Clinically, these findings align with evidence showing that lower DIAPH3 expression is linked to shorter survival in GBM patients and can therefore be considered as a prognostic biomarker [51]. Using this genetically engineered mouse model, along with high-resolution MRI, will provide access to stages of glioma that are not accessible in humans and help understand glioma initiation and progression. However, this model does not necessarily involve the same molecular alterations as in all human glioblastomas, limiting the clinical relevance of the findings.

## Supplementary information

Supplementary Information

Supplementary Table S1

Supplementary Table S2

Supplementary Table S3

Supplementary Table S4

Supplementary File 1

## Data Availability

All the data are included in the main manuscript or as supplementary material. Full and uncropped western blots are provided as Supplementary Material and titled Supplementary File 1.
