# Supplementary material for: Loss of DIAPH3 accelerates glioma genesis in mice
Source: Cell Death Dis. 2026 Mar 23;17(1):342. doi: 10.1038/s41419-026-08652-x (PMC13040077; doi:10.1038/s41419-026-08652-x)
Supplement: Supplementary file 1 — Supplementary Information [file 41419_2026_8652_MOESM1_ESM.docx]

**Loss of DIAPH3 accelerates glioma genesis in mice**.

Georges Chehade^1,6^, Irene Dura Esteve^1,6^, Nuria Ruiz-Reig^1^, Devid Damiani^1,2^, Eva On-Chai Lau^1^, Julie Lelotte^3^, Nicolas Joudiou^4^, Mohamed Aittaleb^5^, Fadel Tissir^1,5✉^

1: Université catholique de Louvain, Institute of Neuroscience, Developmental Neurobiology laboratory, 1200 Brussels, Belgium

2: Current affiliation: Department of Neurobiology and Molecular Medicine, IRCCS Fondazione Stella Maris, 56128 Pisa, Italy

3: Saint-Luc University Hospital, Department of Neuropathology, 1200 Brussels, Belgium, 1200 Brussels, Belgium

4: Université catholique de Louvain, Louvain Drug Research Institute, Nuclear and Electron Spin Technologies Platform, 1200 Brussels, Belgium

5: College of Health and Life Sciences, Hamad Bin Khalifa University, 00000 Doha, Qatar

6: These authors contributed equally

^✉^: Correspondence to Fadel Tissir, Hamad bin Khalifa University, College of Health and Life Sciences, Penrose House Building, Education City, Doha, Qatar

Phone: +974 508 12319

**Email:** [ftissir@hbku.edu.qa](mailto:ftissir@hbku.edu.qa)

**Running Title: DIAPH3 in glioma**

**This file contains supplementary materials and methods, and supplementary figures.**

**Supplementary Materials and Methods**

**Hematoxylin and eosin staining**

Slides were stained with Mayer's hemalum solution (Merck, 109249) for 3 min then differentiated in running tap water for 20 min. This was followed by staining with alcoholic 0.5% eosin Y solution (Merck, 102439) for 10 sec and a wash in running distilled water for 2 min. Slides were dehydrated in isopropanol, incubated three-times in toluene for 3 min then coverslips were mounted with Neo-Mount (Merck, 109016). Images were acquired with a Zeiss Axioskop 40 microscope equipped with a Zeiss Axiocam 305 color camera (Zeiss, Germany).

**In-situ hybridization**

The RNAscope™ 2.5 HD Assay – RED was used to hybridize brain sections with *Diaph3* probe (Bio-Techne, 466231), according to the manufacturer’s protocol. Nuclei were counterstained with DAPI (Merck, D9564) and images were acquired with an Olympus Fluoview FV1000 laser scanning confocal microscope (Olympus, Japan).

**Immunohistochemistry**

After antigen retrieval for 20 min at 90 °C using 0.01 M sodium citrate buffer, pH 6.0, sections were blocked in PBST (0.1% Triton X-100 (Merck, T8787) in PBS supplemented with 3% goat serum (Merck, G9023) and 1% bovine serum albumin (VWR Chemicals, 97061-422) during 1 hour at room temperature. Then they were incubated overnight at 4 °C with the corresponding primary antibodies. The slides were washed and incubated with secondary antibodies for 1 hour at room temperature. The primary antibodies were as follows: mouse anti-MKI67 (BD Biosciences, 556003, 1:50), mouse anti-GFAP (Merck, G3893, 1:500), rabbit anti-OLIG2 (Merck, AB9610, 1:500) and rabbit anti-gamma-H2AX (Abcam, Ab2893, 1:200). Alexa Fluor-conjugated secondary antibodies (1:500) were used, namely, goat anti-mouse Alexa Fluor™ 568 (Invitrogen, A21124) and goat anti-rabbit Alexa Fluor™ 488 (Invitrogen, A11034). The nuclei were counterstained with 100 µM 4',6-diamidino-2-phenylindole (DAPI) (Merck, D9564) for 5 min, and coverslips were then mounted with Mowiol. Images were captured with an Olympus Fluoview FV1000 laser scanning confocal microscope (Olympus, Japan).

**Western blotting**

End-stage high-grade diffuse glioma samples were homogenized on ice in RIPA lysis buffer (Thermo Fisher Scientific, 89900), supplemented with protease/phosphatase inhibitor (Cell Signaling, 5872), using a Bandelin Sonopuls mini20 ultrasonic homogenizer (Bandelin, Germany), and centrifuged at 15000 g for 10 min at 4 °C. Protein quantification was performed with a Qubit 4.0 Fluorometer (Invitrogen, Carlsbad, CA). The supernatant was mixed with 4× NuPAGE™ LDS Sample buffer (Invitrogen, NP0007) and TCEP (Thermo Fisher Scientific, 77720), and heated at 95 °C for 5 min. An equal amount of proteins (15 µg) was loaded on 4-12% Bolt™ Bis-Tris gels (Thermo Fisher Scientific, NW04125), separated by NuPAGE™ MES SDS running buffer (Thermo Fisher Scientific, NP0002) or Bolt™ MOPS SDS running buffer (Thermo Fisher Scientific, B0001), then transferred to PVDF membranes (Merck, ISEQ00005) by NuPAGE™ transfer buffer (Thermo Fisher Scientific, NP00061), supplemented with 15% methanol (70 V for 1.5 h at 4 °C). Membranes were blocked for 1 h at room temperature in TBST (0.1% Tween-20 (VWR Chemicals, 97062-332) in tris-buffered saline), supplemented with 5% fat-free milk (Merck, 70166), and incubated overnight at 4 °C with one of the following primary antibodies: rabbit anti-γ-H2AX (Abcam, Ab2893, 1:1000), chicken anti-GAPDH (Merck, AB2302, 1:2000), and rabbit anti-FGFR2 (Cell Signaling, 23328, 1:1000). Membranes were then incubated with horseradish peroxidase-coupled secondary antibodies (1:20000): goat anti-rabbit (Cell Signaling, 7074), and rabbit anti-chicken (Merck, 12-341). Membranes were revealed using SuperSignal™ West Pico PLUS chemiluminescent substrate (Thermo Fisher Scientific, 34577) or SuperSignal™ West Femto Maximum sensitivity substrate (Thermo Fisher Scientific, 34095), and imaged using Fusion Pulse system (Vilber, France) and analyzed in Fiji software (ImageJ). Values were normalized to GAPDH.

**RNA sequencing and analysis**

Total RNA was extracted from the olfactory bulbs (OB) of 4-months-old mice in a pretumoral stage or from end-stage high-grade diffuse gliomas using using the RNeasy Mini Kit (Qiagen, 74104) according to the manufacturer’s instructions. RNA concentration was determined using Qubit 4.0 Fluorometer (Invitrogen, Carlsbad, CA) and RNA integrity was evaluated via the RNA Kit on Agilent 5300 Fragment Analyzer (Agilent Technologies, Palo Alto, CA, USA). For library transcriptome preparation the NEBNext® Ultra™ II RNA Library Prep Kit for Illumina was used in accordance with the manufacturer’s guidelines (NEB, Ipswich, MA, USA). In brief, mRNAs were first enriched with Oligo(dT) beads and followed by fragmentation. First strand and second strand cDNAs were subsequently synthesized. The resulting cDNA fragments underwent end-repair and 3’ adenylation. Universal adapters were ligated to cDNA fragments and followed by index addition and library enrichment through limited-cycle PCR. Sequencing libraries were validated using NGS Kit on the Agilent 5300 Fragment Analyzer (Agilent Technologies, Palo Alto, CA, USA), and concentrations were determined by using Qubit 4.0 Fluorometer (Invitrogen, Carlsbad, CA). The sequencing libraries were multiplexed and loaded onto the Illumina NovaSeq X Plus. Samples were sequenced using a 2×150 Pair-End configuration v1.5. Image analysis and base calling were performed using the NovaSeq Control Software v1.7. The resulting raw sequence data (.bcl files) were converted into FASTQ format and demultiplexed using Illumina bcl2fastq program version 2.20 allowing a maximum of one mismatch per barcode. To ensure data quality, sequencing reads were trimmed to remove possible adapter sequences and nucleotides with poor quality using Trimmomatic v.0.36. Clean reads were then aligned to the Mus musculus ENSEMBL reference genome using STAR aligner v.2.5.2b. Unique gene hit counts were calculated using feature *Counts* from the Subread package v.1.5.2, counting only unique reads included in exon regions. Subsequently, gene count tables were generated for downstream expression profiling. Differential expression analysis was conducted using DESeq2. Statistical significance was determined using the Wald test, generating both *P* values and log2 (fold changes). Genes were considered differentially expressed if they had an adjusted *P* value < 0.05 and absolute log2 (fold changes) > 0 for pre-tumoral stages and > 1 for end stage glioma. GO and KEGG graphs were performed using all expressed genes as background for enrichment analysis. For both analyses, cluster Profiler package was used on the principle of hypergeometric distribution.

**Shallow whole-genome sequencing**

For the DNA library preparation and NovaSeq sequencing, total DNA was extracted from end-stage gliomas using a QIAamp DNA Mini Kit (Qiagen, 51304). DNA samples were quantified using the Qubit 4.0 Fluorometer (Invitrogen, Carlsbad, CA) and qualified using the Agilent 5600 Fragment Analyzer (Agilent Technologies, Palo Alto, CA, USA). Genomic DNA (100 ng) was fragmented, end-repaired, and adenylated. Adapters were ligated after adenylation of the 3’ ends followed by enrichment by limited cycle PCR. The DNA libraries were validated using the Agilent 5600 Fragment Analyzer (Agilent Technologies, Palo Alto, CA, USA). Quantification was performed using Qubit 4.0 Fluorometer (Invitrogen, Carlsbad, CA). The libraries were multiplexed on a flow cell and loaded onto the Illumina NovaSeq 6000. Samples were sequenced using a 2×150 paired-end configuration. Image analysis and base calling were conducted with the NovaSeq Control Software on the NovaSeq instrument. Finally, raw sequencing data generated from Illumina NovaSeq were converted into FASTQ files and demultiplexed with Illumina's bcl2fastq software.

For the data analysis, we aligned raw data (.fastq) files to the reference mouse genome assembly (GRCm38) using BWA 0.7.15. CNAs were called using QDNAseq with bins of 30K bases and annotated using AnnotSV 2.5.

**Quantitative reverse transcription PCR**

Total RNA was isolated from end-stage diffuse glioma samples utilising RNeasy Mini Kit (Qiagen, Cat# 74104). RNA concentration was measured with a Qubit 4.0 Fluorometer (Invitrogen, Carlsbad, CA). cDNA synthesis was carried out using a GoScript™ Reverse Transcription Mix, Random Primers (Promega, A2801). We proceeded with quantitative PCR using iQ™ SYBR® Green Supermix (Cat# 1708882) with a CFX96 Touch real-time PCR detection system (Bio-Rad). *Gapdh* and *Actb* were used as housekeeping genes to normalize RNA expression. Relative expressions were computed using the Pffafl method.

**Statistical analysis**

Sample size was determined based on previous literature (1, 2) examining similar interventions to achieve at least α = 0.05 and 90% power. No data points, samples or animals were removed or excluded at any stage of the analysis. No randomization or blinding method was implemented during the experiments. Survival data was analyzed by Kaplan‒Meier curves and then their statistical differences were compared using the log-rank test. Comparisons of categorical variables were made using Pearson’s chi-squared test, Fisher’s exact test or the likelihood-ratio test depending on the applicability of each. Continuous variables were assessed using either the independent-samples *t* test or Mann‒Whitney test after verification of the normality of the distribution by the Kolmogorov‒Smirnov test and Shapiro‒Wilk test. Homogeneity of variance was tested before performing the statistical analyses. Variances were similar between groups. Unquantified observations were validated in at least three different individuals. Statistical analyses were conducted using IBM SPSS and graphs were created using GraphPad Prism. n.s., not significant; *, *P* < 0.05; **, *P* < 0.01; ***, *P* < 0.001. The center values, standard errors of the means (SEMs), 95% confidence intervals (CIs), sample sizes, *P* values and statistical tests used are detailed in each figure and table legend.

**Supplementary figures and captions**


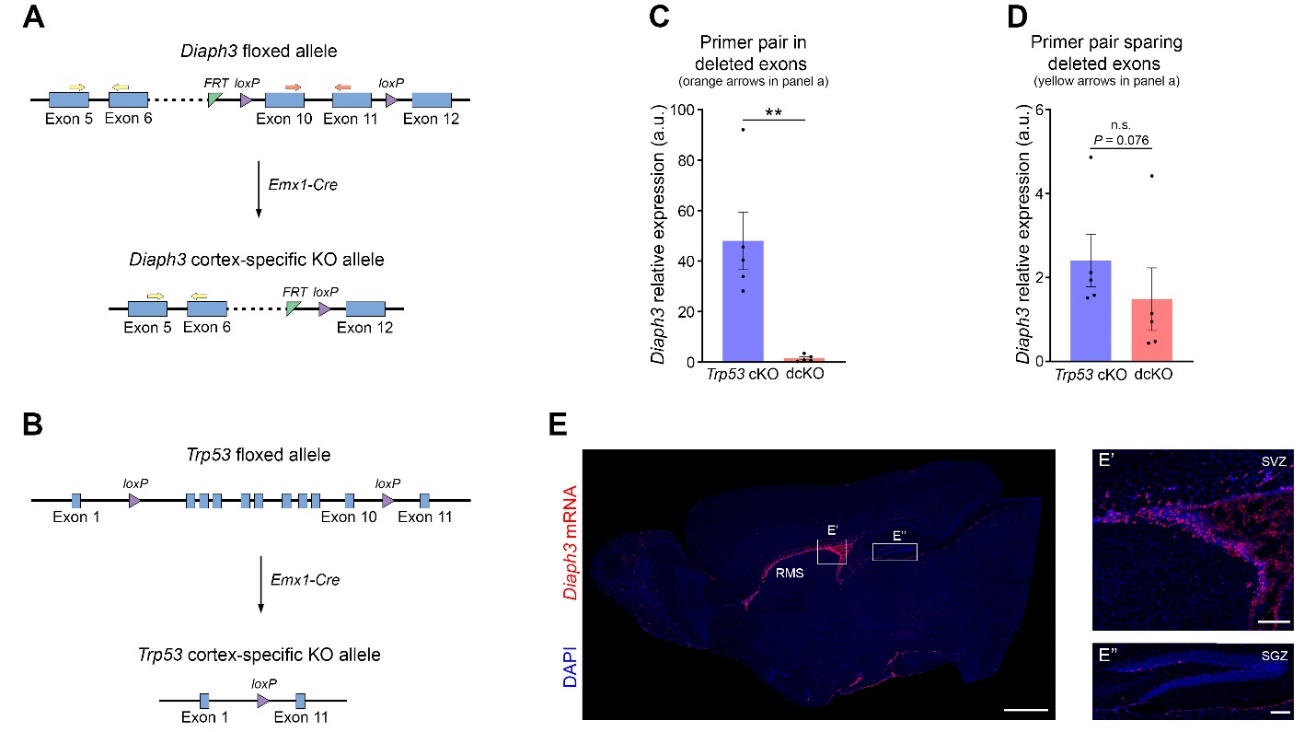


**Supplementary Fig. S1. DIAPH3 loss in dcKO high-grade diffuse gliomas.** (A, B) Schematic diagrams illustrating the *Diaph3* and the *Trp53* floxed alleles as well as the corresponding cortex-specific knockout (KO) alleles. (C, D) *Diaph3* relative expression in diffuse gliomas measured by quantitative reverse transcription PCR. The primers used for the floxed/deleted exons were as follows: forward primer TGCTCTCGTTACATCTCCTGA, reverse primer TGTCTTCAAGGCGATGGAAA (*Trp53* cKO: mean = 48.037 a.u., SEM = 11.405, *n* = 5; dcKO: mean = 1.447 a.u., SEM = 0.587, *n* = 5; *P* = 0.009 by Mann-Whitney test; C). The primers used for unaffected exons 5 and 6 were the following: forward primer CCGACAGATCTCACCTCAGG, reverse primer CCAGCAATAATCCGAGTCCC (*Trp53* cKO: mean = 2.406 a.u., SEM = 0.626, *n* = 5; dcKO: mean = 1.486 a.u., SEM = 0.746, *n* = 5; *P* = 0.076 by Mann-Whitney test, D). *Gapdh* (forward primer AGGTCGGTGTGAACGGATTTG, reverse primer TGTAGACCATGTAGTTGAGGTCA) and *Actb* (forward primer GGAAAAGAGCCTCAGGGCAT, reverse primer GAAGAGCTATGAGCTGCCTGA) were used as internal controls. (E) Adult wild-type mouse brain section hybridized with *Diaph3* (red) RNAscope™ probe. Nuclei were counterstained with DAPI (blue). (E’, E”) High magnification of the subventricular zone (SVZ) (E’) and subgranular zone (SGZ) (E”). Scale bars: (E) 1 mm; (E’, E”) 100 µm. Abbreviations: KO, knockout; RMS, rostral migratory stream; SVZ, subventricular zone; SGZ, subgranular zone.


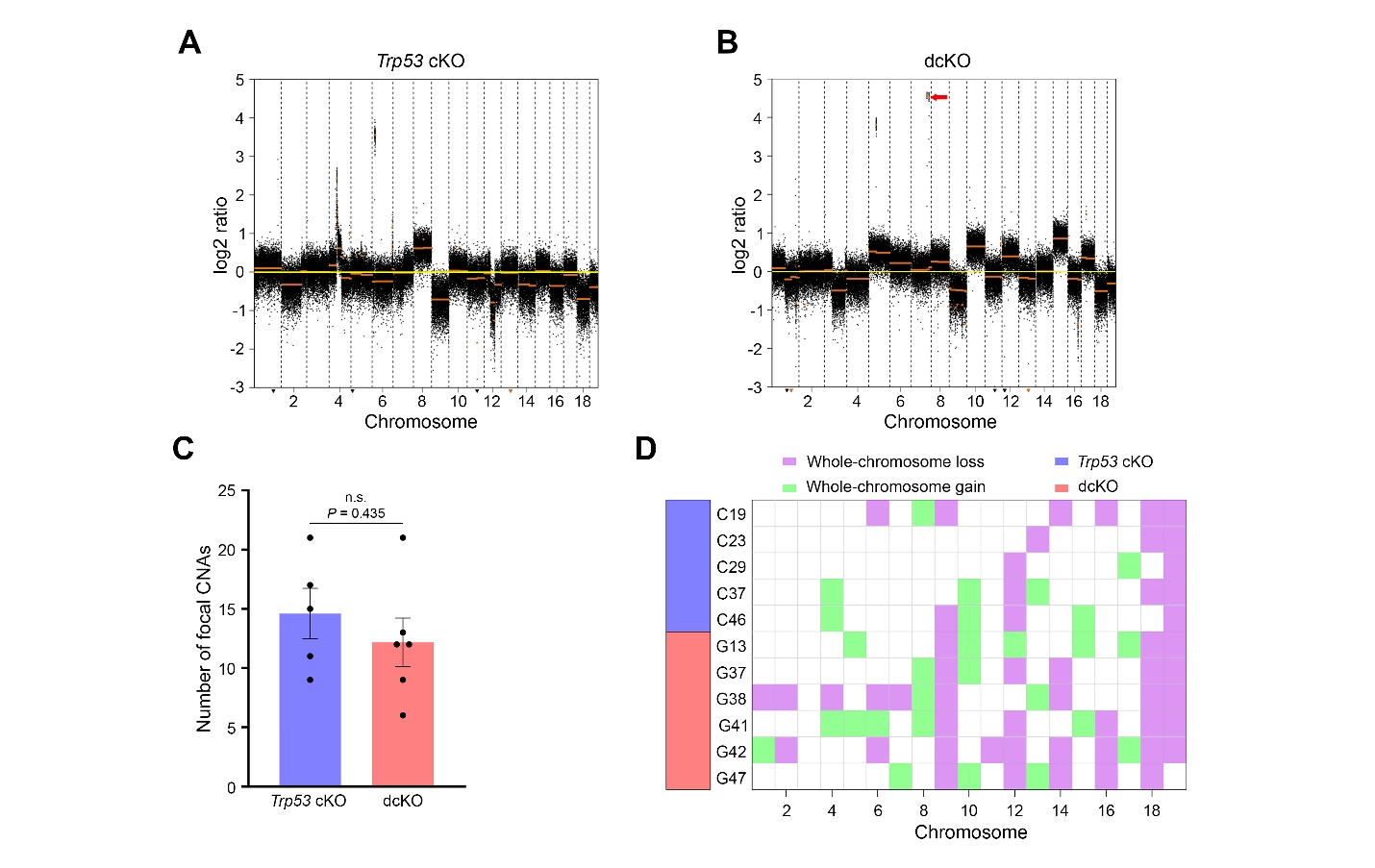
 **Supplementary** **Fig. S2. Copy number alteration analysis.** (A, B) Representative copy number alteration (CNA) plots for *Trp53* cKO (A) and dcKO (B) high-grade diffuse gliomas. The red arrow points to the high-level amplification of *Fgfr2* in dcKO (B). (C) Number of focal CNAs in *Trp53* cKO and dcKO high-grade diffuse gliomas (*Trp53* cKO: mean = 14.600 chromosomes, SEM = 2.135, *n* = 5; dcKO: mean = 12.167 chromosomes, SEM = 2.056, *n* = 6; *P* = 0.435 by independent-samples *t* test). (D) Schematic representation of the whole-chromosome gain (green) and loss (magenta) in *Trp53* cKO (*n* = 5) and dcKO (*n* = 6). Abbreviations: CNA, copy number alteration.


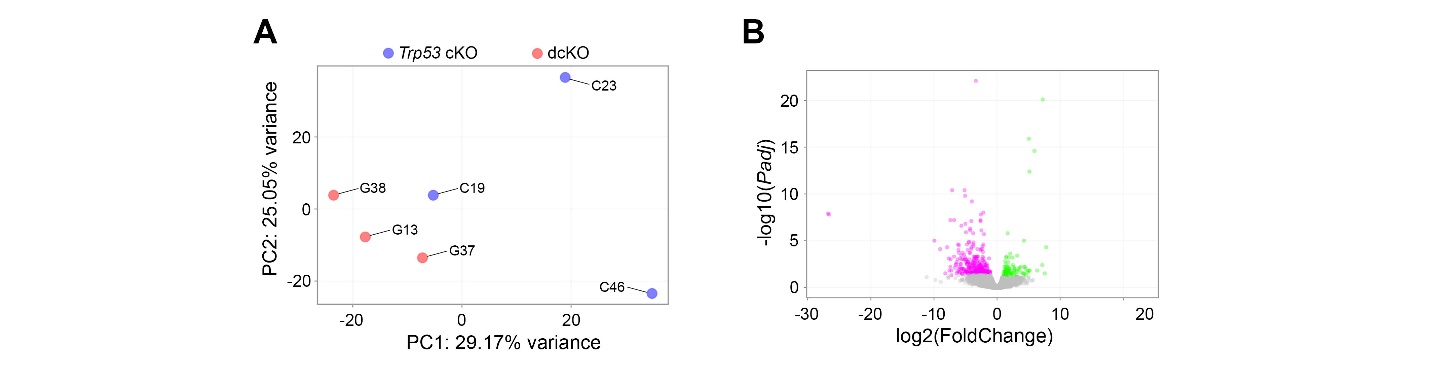
**Supplementary Fig. S3. Transcriptomic analysis of Trp53 cKO and dcKO high-grade diffuse gliomas.** (A) Principal component analysis showing overall differences between *Trp53* cKO and dcKO diffuse glioma samples, based on RNA sequencing data (*n* = 3 for each genotype). (B) Volcano plot showing upregulated (green) and downregulated (magenta) genes in dcKO compared to *Trp53* cKO diffuse gliomas.
