# Supplementary material for: Loss of DIAPH3 accelerates glioma genesis in mice
Source: Cell Death Dis. 2026 Mar 23;17(1):342. doi: 10.1038/s41419-026-08652-x (PMC13040077; doi:10.1038/s41419-026-08652-x)
Supplement: Supplementary file 4 — Supplementary Table S3 [file 41419_2026_8652_MOESM4_ESM.docx]

**Supplementary Table S3: List of DEGs involved in cancer, immunity, angiogenesis or invasiveness.**

| **Gene Name** | **Change** | **Cancer** | **Immunity** | **Angiogenesis** | **ECM** |
| --- | --- | --- | --- | --- | --- |
| Muc3a | Up | (3) |  |  | (4) |
| Prkcg | Down | (5) |  |  |  |
| Psmb5 | Up | (6) | (7) |  |  |
| Tafa1 | Down |  | (8) |  |  |
| Serpina3n | Up | (9) |  |  |  |
| Klhl33 | Down | (10) |  |  |  |
| Timp3 | Up |  |  |  | (11) |
| Fkbp3 | Down | (12) | (13) |  |  |
| Setd3 | Down | (14) |  |  |  |
| Thsd4 | Up | (15) |  |  | (16) |
| Rgs14 | Down | (17) |  |  |  |
| Tpbg | Up | (18) |  |  |  |
| Dbpht2 | Down |  |  |  |  |
| Rnf112 | Down | (19) |  |  |  |
| Slc6a16 | Up | (20) |  |  |  |
| Scube2 | Up | (21) |  |  |  |
| Gnpda1 | Down | (22) |  |  |  |
| Cthrc1 | Up | (23) |  | (24) |  |
| Tmem249 | Down | (25) |  |  |  |
| Ppp1r3e | Up | (26) |  |  |  |
| Zfp772 | Down |  |  |  |  |
| Ston1 | Up | (27) |  |  |  |
| Pcdh17 | Up | (28) |  |  |  |
| Mpped1 | Down | (29) |  |  |  |
| Aldh1a1 | Up | (30) |  | (31) |  |
| Slc7a5 | Up | (32) |  |  |  |
| Cd6 | Down | (33) | (34) |  |  |
| Fmnl1 | Down | (35) |  |  |  |
| Htr2c | Down | (36) |  |  |  |
| Deptor | Up | (37) |  | (37) |  |
| Pcdh9 | Up | (38) |  |  |  |
| Tmem215 | Down | (39) |  | (40) |  |
| Ly6a | Up | (41) | (42) |  |  |
| Htr1b | Down | (43) |  |  |  |
| Sc5d | Down | (44) |  |  |  |
| Rnf26 | Down | (45) |  |  |  |
| Rgs4 | Down | (46) |  |  |  |
| Faah | Down | (47) |  |  |  |
| C4b | Up | (48) | (49) |  |  |
| Kcnn2 | Up | (50) |  |  |  |
| Lmo3 | Down | (51) |  |  |  |
| Mal2 | Down | (52) |  |  |  |
| Cnr1 | Down | (53) |  |  |  |
| Tmc4 | Down | (54) |  |  |  |
| H2-T22 | Down |  | (55) |  |  |
| Mas1 | Down | (56) |  |  |  |
| Slc41a1 | Up | (57) |  |  |  |
| Serpine1 | Up | (58) |  |  |  |
| Emc10 | Down | (59) |  | (60) |  |
| Nrgn | Down | (61) |  |  |  |
| Pnp2 | Up | (62) | (63) |  |  |
| Crb2 | Down | (64) |  |  |  |
| Shisa3 | Up | (65) |  |  |  |
| Rit2 | Down | (66) |  |  |  |
| Rps6-ps4 | Down |  |  |  |  |
| Mrap2 | Up | (67) |  |  |  |
| Adipor2 | Up | (68) |  |  |  |
| Tlr6 | Down | (69) | (70) |  |  |
| Josd2 | Down | (71) |  |  |  |
| Atp6v0e2 | Down | (72) |  |  |  |
| Kitl | Down | (73) |  |  |  |
| Ccnb1ip1 | Down | (74) |  |  |  |
| Col26a1 | Down | (75) | (76) |  |  |
| Trim56 | Up | (77) |  |  |  |
| Glt8d2 | Down | (78) |  |  |  |
| Luc7l3 | Down | (79) |  |  |  |

**Supplementary references**

1. Lau EO, Damiani D, Chehade G, Ruiz-Reig N, Saade R, Jossin Y, et al. DIAPH3 deficiency links microtubules to mitotic errors, defective neurogenesis, and brain dysfunction. Elife. 2021;10.

2. Ruiz-Reig N, Chehade G, Hakanen J, Aittaleb M, Wierda K, De Wit J, et al. KIF2A deficiency causes early-onset neurodegeneration. Proc Natl Acad Sci U S A. 2022;119(46):e2209714119.

3. Bhattacharya M, Yaniv D, D'Souza DP, Yosefof E, Tzelnick S, Detroja R, et al. Applications for Circulating Cell-Free DNA in Oral Squamous Cell Carcinoma: A Non-Invasive Approach for Detecting Structural Variants, Fusions, and Oncoviruses. Cancers (Basel). 2025;17(12).

4. Williams SJ, Munster DJ, Quin RJ, Gotley DC, McGuckin MA. The MUC3 gene encodes a transmembrane mucin and is alternatively spliced. Biochem Biophys Res Commun. 1999;261(1):83-9.

5. Dowling CM, Hayes SL, Phelan JJ, Cathcart MC, Finn SP, Mehigan B, et al. Expression of protein kinase C gamma promotes cell migration in colon cancer. Oncotarget. 2017;8(42):72096-107.

6. Tang D, Lin S, Zhou J, Lei JH, Shao F, Sun H, et al. Augment proteasome inhibitor efficacy activates CD8(+) T cell-mediated antitumor immunity in breast cancer. Cell Rep Med. 2025;6(7):102211.

7. Shinebaatar E, Morimoto J, Koga R, Nguyen TN, Sasaki Y, Yonemura S, et al. Proteasome dysfunction in T cells causes immunodeficiency via cell cycle disruption and apoptosis. Int Immunol. 2025;37(8):493-505.

8. Sarver DC, Lei X, Wong GW. FAM19A (TAFA): An Emerging Family of Neurokines with Diverse Functions in the Central and Peripheral Nervous System. ACS Chem Neurosci. 2021;12(6):945-58.

9. Wierer M, Verde G, Pisano P, Molina H, Font-Mateu J, Di Croce L, et al. PLK1 signaling in breast cancer cells cooperates with estrogen receptor-dependent gene transcription. Cell Rep. 2013;3(6):2021-32.

10. Li J, Zeng J, Luo S, Wang J. Molecular genetic analysis of pulmonary benign metastasizing leiomyoma and intravenous leiomyomatosis: a comparative study using whole exome sequencing. Discov Oncol. 2025;16(1):939.

11. Li Y, Zhang T, Tian W, Hu H, Xin Z, Ma X, et al. Loss of TIMP3 expression induces inflammation, matrix degradation, and vascular ingrowth in nucleus pulposus: A new mechanism of intervertebral disc degeneration. FASEB J. 2020;34(4):5483-98.

12. Zhang Y, Chen F, Cao Y, Zhang H, Zhao L, Xu Y. Identifying diagnostic markers and establishing prognostic model for lung cancer based on lung cancer-derived exosomal genes. Cancer Biomark. 2025;42(2):18758592251317400.

13. Li S, Yang L, Li J. FKBP3, a poor prognostic indicator, promotes the progression of LUAD via regulating ferroptosis and immune infiltration. Medicine (Baltimore). 2024;103(26):e38606.

14. Wu W, Wu W, Zhou Y, Yang Q, Zhuang S, Zhong C, et al. The dePARylase NUDT16 promotes radiation resistance of cancer cells by blocking SETD3 for degradation via reversing its ADP-ribosylation. J Biol Chem. 2024;300(3):105671.

15. Liu J, Huang Z, Chen HN, Qin S, Chen Y, Jiang J, et al. ZNF37A promotes tumor metastasis through transcriptional control of THSD4/TGF-beta axis in colorectal cancer. Oncogene. 2021;40(19):3394-407.

16. Wang M, Wang M, Jiang J, Li K, Liang H, Wang N, et al. THSD4 promotes hair growth by facilitating dermal papilla and hair matrix interactions. Theranostics. 2025;15(8):3571-88.

17. Liang X, Xu B, Wang Q, Gong K, Han C, Sun B, et al. RGS14 promotes the progression of hepatocellular carcinoma by activating the cAMP/PKA/CREB signaling pathway. J Cancer Res Clin Oncol. 2025;151(5):153.

18. Su H, Yu S, Sun F, Lin D, Liu P, Zhao L. LINC00342 induces metastasis of lung adenocarcinoma by targeting miR-15b/TPBG. Acta Biochim Pol. 2022;69(2):291-7.

19. Xiong K, Chen S, Xu H, Tu S, Weng H, Wang Y, et al. RNF112 Facilitates Ubiquitin-Mediated Degradation of c-Myc, Suppressing Proliferation, Migration and Lipid Synthesis in Bladder Cancer. Adv Sci (Weinh). 2025;12(20):e2408311.

20. Park SJ, Kim H, Kim SH, Joe EH, Jou I. Epigenetic downregulation of STAT6 increases HIF-1alpha expression via mTOR/S6K/S6, leading to enhanced hypoxic viability of glioma cells. Acta Neuropathol Commun. 2019;7(1):149.

21. Khorshid Sokhangouy S, Zeinali M, Fathi S, Nazari E. Deep learning assisted identification of SCUBE2 and SLC16 A5 combination in RNA-sequencing data as a novel specific potential diagnostic biomarker in prostate cancer. Med Biol Eng Comput. 2025.

22. Liu P, Yang D, Ma R. Overexpression of GNPDA1 in head and neck squamous cell carcinoma: Prognostic significance, immune infiltration, and correlation with cancer cell immune evasion. Medicine (Baltimore). 2025;104(21):e42561.

23. Yin H, Pan Y, Li Z, Liu Y, Chen J, Chen X, et al. CTHRC1 Derived From Cancer-Associated Fibroblasts Promotes Pancreatic Cancer Progression and Metastasis via the LIF-STAT3 Pathway. Cancer Med. 2025;14(15):e71126.

24. Wang M, Hu H, Xiao H, Wang Y, Hao L, Cao Y. CTHRC1 promotes hepatocellular carcinoma proliferation, migration and invasion by regulating VEGF expression and validation of MRI images. Technol Health Care. 2025:9287329251356944.

25. Sharbatoghli M, Fattahi F, Aboulkheyr Es H, Akbari A, Akhavan S, Ebrahimi M, et al. Copy Number Variation of Circulating Tumor DNA (ctDNA) Detected Using NIPT in Neoadjuvant Chemotherapy-Treated Ovarian Cancer Patients. Front Genet. 2022;13:938985.

26. Zhao Y, Zhang B, Ma Y, Guo M, Zhao F, Chen J, et al. Distinct molecular profiles drive multifaceted characteristics of colorectal cancer metastatic seeds. J Exp Med. 2024;221(5).

27. Zheng A, Bai J, Ha Y, Yu Y, Fan Y, Liang M, et al. Integrated analysis of the relation to tumor immune microenvironment and predicted value of Stonin1 gene for immune checkpoint blockage and targeted treatment in kidney renal clear cell carcinoma. BMC Cancer. 2023;23(1):135.

28. Sun Y, Wan H, Xiong J, Cao K, Yang D, Huang J. Single-cell and transcriptomic analyses reveal the role of PCDH17 in the non-inflammatory tumor microenvironment of pancreatic cancer. Front Endocrinol (Lausanne). 2025;16:1559909.

29. Li S, Liu X, Zhou Y, Acharya A, Savkovic V, Xu C, et al. Shared genetic and epigenetic mechanisms between chronic periodontitis and oral squamous cell carcinoma. Oral Oncol. 2018;86:216-24.

30. Wang X, Wen X, Hu X, Niu W, Guo G, Yang F, et al. ALDH1A1 promotes colorectal cancer metastasis through activating the notch signaling pathway. Med Oncol. 2025;42(9):403.

31. Ciccone V, Terzuoli E, Ristori E, Filippelli A, Ziche M, Morbidelli L, et al. ALDH1A1 overexpression in melanoma cells promotes tumor angiogenesis by activating the IL‑8/Notch signaling cascade. Int J Mol Med. 2022;50(1).

32. Hendi NN, Nemer G. SDR42E1 modulates vitamin D absorption and cancer pathogenesis: insights from an in vitro model. Front Endocrinol (Lausanne). 2025;16:1585859.

33. Sanvicente A, Nieto-Jimenez C, Morafraile EC, Diaz-Tejeiro C, Barberan VG, Segura PP, et al. Identification of Deregulated Proteins in Mutated BRCA1/2 Breast and Ovarian Cancers for Vectorized Biologics. Cancers (Basel). 2025;17(13).

34. Santos RF, de Sousa Linhares A, Steinberger P, Davis SJ, Oliveira L, Carmo AM. The CD6 interactome orchestrates ligand-independent T cell inhibitory signaling. Cell Commun Signal. 2024;22(1):286.

35. Zhang MF, Li QL, Yang YF, Cao Y, Zhang CZ. FMNL1 Exhibits Pro-Metastatic Activity via CXCR2 in Clear Cell Renal Cell Carcinoma. Front Oncol. 2020;10:564614.

36. Zhan D, Wang X, Zheng Y, Wang S, Yang B, Pan B, et al. Integrative dissection of 5-hydroxytryptamine receptors-related signature in the prognosis and immune microenvironment of breast cancer. Front Oncol. 2023;13:1147189.

37. Xiong X, Chen X, Shao S, Cui D, Qu R, Wang B, et al. DEPTOR suppresses lymphomagenesis by promoting EGFR degradation via HUWE1 E3 ligase. Cell Death Differ. 2025.

38. Li A, Sun W, Shao S, Qiu X, Hu J, Cui F. The lncRNA MIR22HG suppresses prostate cancer cell proliferation, migration, and epithelial-mesenchymal transition via the miR-4428/PCDH9 axis. Transl Cancer Res. 2025;14(5):3133-48.

39. Hajebi Khaniki S, Shokoohi F. Data-Driven Identification of Early Cancer-Associated Genes via Penalized Trans-Dimensional Hidden Markov Models. Biomolecules. 2025;15(2).

40. Zhang P, Yan X, Zhang X, Liu Y, Feng X, Yang Z, et al. TMEM215 Prevents Endothelial Cell Apoptosis in Vessel Regression by Blunting BIK-Regulated ER-to-Mitochondrial Ca Influx. Circ Res. 2023;133(9):739-57.

41. Cao L, Li Y, Ou M, Smirnov A, Liu R, Wang T, et al. Mesenchymal stromal cells highly expressing Sca-1 promote breast cancer lung metastasis through recruiting myeloid cells. Cell Death Dis. 2025;16(1):507.

42. Goswami M, Celades C, Minnar CM, Khelifa AS, Poppe LK, Bracken-Clarke D, et al. Increased peripheral T stem cell-like memory features in patients with advanced solid tumors treated with tumor-targeting IL-12 immunocytokine therapy. Clin Cancer Res. 2025.

43. Gurban P, Mambet C, Botezatu A, Necula LG, Matei L, Neagu AI, et al. Increased mRNA expression for serotonin receptor 1B (HTR1B) is associated with thrombosis in BCR::ABL1-negative myeloproliferative neoplasms. J Cell Mol Med. 2024;28(16):e70024.

44. Mironov A, Franchitti L, Ghosh S, Ritz MF, Hutter G, De Bortoli M, et al. Leveraging multi-omics data to infer regulators of mRNA 3' end processing in glioblastoma. Front Mol Biosci. 2024;11:1363933.

45. Yao D, Xin F, He X. RNF26-mediated ubiquitination of TRIM21 promotes bladder cancer progression. Am J Cancer Res. 2024;14(8):4082-95.

46. Wang X, Zhang W, Liang K, Wang Y, Zhang J, Wang J, et al. Identification of m6 A-regulated ferroptosis biomarkers for prognosis in laryngeal cancer. BMC Cancer. 2025;25(1):694.

47. Schirizzi A, Renna N, De Leonardis G, Montanaro R, Mastropasqua F, Graziano G, et al. CC48 a new CB2R agonist/FAAH inhibitor dual drug blocks gastric cancer progression and overcomes paclitaxel resistance. J Exp Clin Cancer Res. 2025;44(1):209.

48. Akbarzadeh T, Ma L, Lee J, Moore J, Chou W, Shabani S, et al. Plasma proteomic analysis reveals complement system changes in irradiated female BALB/c mice during mammary carcinogenesis. Cancer Res Commun. 2025.

49. Kadava T, Strasser J, Marefat M, Yin VC, Preiner J, Trouw LA, et al. Structural Insights into Complement Inhibition: Visualizing Distinct Binding Modes of C4b-binding Protein Complexes with C4b and SAP. Mol Cell Proteomics. 2025:101046.

50. Gao Y, Chen J, Du W. Identification of novel potential biomarkers using bulk RNA and single cells to build a neural network model for diagnosis of liver cancer. Discov Oncol. 2025;16(1):728.

51. Ling Z, Long X, Wu Y, Li J, Feng M. LMO3 promotes proliferation and metastasis of papillary thyroid carcinoma cells by regulating LIMK1-mediated cofilin and the beta-catenin pathway. Open Med (Wars). 2022;17(1):453-62.

52. Davis SS, Bassaro LR, Tuma PL. MAL2 and rab17 selectively redistribute invadopodia proteins to laterally-induced protrusions in hepatocellular carcinoma cells. Mol Biol Cell. 2025;36(3):ar26.

53. Ye Y, Zhong W, Liang L, Han R, Han J, Wang C, et al. Assessing the toxicological impact of DEGDB plasticizer exposure on glioblastoma multiforme via network toxicology, machine learning and in vitro methods. Environ Pollut. 2025;383:126843.

54. Tang W, Shi Z, Zhu Y, Shan Z, Jiang A, Wang A, et al. Comprehensive analysis of the prognosis and immune infiltration of TMC family members in renal clear cell carcinoma. Sci Rep. 2023;13(1):11668.

55. Moriwaki S, Korn BS, Ichikawa Y, van Kaer L, Tonegawa S. Amino acid substitutions in the floor of the putative antigen-binding site of H-2T22 affect recognition by a gamma delta T-cell receptor. Proc Natl Acad Sci U S A. 1993;90(23):11396-400.

56. Luo Y, Tanabe E, Kitayoshi M, Nishiguchi Y, Fujiwara R, Matsushima S, et al. Expression of MAS1 in breast cancer. Cancer Sci. 2015;106(9):1240-8.

57. Chen G, Du Z, Rao C. SLC41A1 overexpression correlates with immune cell infiltration in HCC and promotes its malignant progression. Int J Med Sci. 2024;21(15):3069-82.

58. Chen S, Li Y, Zhu Y, Fei J, Song L, Sun G, et al. SERPINE1 Overexpression Promotes Malignant Progression and Poor Prognosis of Gastric Cancer. J Oncol. 2022;2022:2647825.

59. Junes-Gill KS, Lawrence CE, Wheeler CJ, Cordner R, Gill TG, Mar V, et al. Human Hematopoietic Signal peptide-containing Secreted 1 (hHSS1) modulates genes and pathways in glioma: implications for the regulation of tumorigenicity and angiogenesis. BMC Cancer. 2014;14:920.

60. Reboll MR, Korf-Klingebiel M, Klede S, Polten F, Brinkmann E, Reimann I, et al. EMC10 (Endoplasmic Reticulum Membrane Protein Complex Subunit 10) Is a Bone Marrow-Derived Angiogenic Growth Factor Promoting Tissue Repair After Myocardial Infarction. Circulation. 2017;136(19):1809-23.

61. Ye D, Zhang Z, Yao Y, Pan B, Wu H, Zhang X, et al. Neurogranin facilitates maintaining the immunosuppressive state of hepatocellular carcinoma by promoting TGF-beta1 secretion. Int J Biol Macromol. 2025;311(Pt 1):143716.

62. Lv X, Chen J, Abuduhailili X, Deng Y, Yuan X, Feng Y. VEGFA and APOE regulate distinct functional states of mast cells in hepatocellular carcinoma: A single-cell transcriptome analysis. Int J Biol Macromol. 2025;321(Pt 1):146131.

63. Wedel J, Kochupurakkal N, Kong SW, Bose S, Lee JW, Maslyar M, et al. Neuropilin-2 functions as a coinhibitory receptor to regulate antigen-induced inflammation and allograft rejection. J Clin Invest. 2025;135(13).

64. Wang Y, Bao G, Zhang M, Xiang J, Zhou H, Wahafu A, et al. CRB2 enhances malignancy of glioblastoma via activation of the NF-kappaB pathway. Exp Cell Res. 2022;414(1):113077.

65. Zhang S, Yu B, Sheng C, Yao C, Liu Y, Wang J, et al. SHISA3 Reprograms Tumor-Associated Macrophages Toward an Antitumoral Phenotype and Enhances Cancer Immunotherapy. Adv Sci (Weinh). 2024;11(36):e2403019.

66. Uenaka T, Satake W, Cha PC, Hayakawa H, Baba K, Jiang S, et al. In silico drug screening by using genome-wide association study data repurposed dabrafenib, an anti-melanoma drug, for Parkinson's disease. Hum Mol Genet. 2018;27(22):3974-85.

67. Berruien NNA, Smith CL. Emerging roles of melanocortin receptor accessory proteins (MRAP and MRAP2) in physiology and pathophysiology. Gene. 2020;757:144949.

68. Ray I, Moller-Levet CS, Michael A, Butler-Manuel S, Chatterjee J, Tailor A, et al. Exploring the Relationship Between Adipocytokines and Endometrial Cancer: Identifying Correlations With Clinico-Pathological Prognostic Factors. Cancer Med. 2025;14(13):e71007.

69. Ma L, Yin Y, Yu Z, Xu N, Ma L, Qiao W, et al. Toll-like receptor 6 inhibits colorectal cancer progression by suppressing NF-kappaB signaling. Heliyon. 2024;10(6):e26984.

70. Mansouri A, Akthar I, Miyamoto A. TLR2 and TLR4 bridge physiological and pathological inflammation in the reproductive system. Commun Biol. 2025;8(1):1008.

71. Yuan T, Liu Y, Wu R, Qian M, Wang W, Li Y, et al. Josephin Domain Containing 2 (JOSD2) inhibition as Pan-KRAS-mutation-targeting strategy for colorectal cancer. Nat Commun. 2025;16(1):3623.

72. Yuan Y, Zhang ZG, Ma B, Ji P, Ma S, Qi X. Effective oxygen metabolism-based prognostic signature for colorectal cancer. Front Oncol. 2023;13:1072941.

73. Onate MK, Oon C, Bhattacharyya S, Low V, Chen C, Zhao X, et al. Stromal KITL/SCF Maintains Pancreas Tissue Homeostasis and Restrains Tumor Progression. Cancer Discov. 2025;15(5):913-29.

74. Liu W, Liang P, Chen L, Liang R, Xiong X. Regulatory RNA network mediated by FUBP1 drives the proliferation and invasion of triple-negative breast cancer cells. Med Oncol. 2025;42(8):293.

75. Fan YW, Liu MH, Xu TJ, Fan RY, Xiang J, Wu JQ, et al. Mechanism of etoposide resistance in small cell lung cancer and the potential therapeutic options. Med Oncol. 2025;42(5):167.

76. Luo Y, Ye Y, Zhang Y, Chen L, Qu X, Yi N, et al. New insights into COL26A1 in thyroid carcinoma: prognostic prediction, functional characterization, immunological drug target and ceRNA network. Transl Cancer Res. 2023;12(12):3384-408.

77. Jiang C, Li H, Li D, Li X, Luo S, You L. The oncogenic role of TRIM56 in pancreatic cancer via the TRAF6/NF-kB axis. J Mol Histol. 2025;56(4):205.

78. Li W, Zuo K, Zhao Q, Guo C, Liu Z, Liu C, et al. An 11-gene glycosyltransferases-related model for the prognosis of patients with bladder urothelial carcinoma: development and validation based on TCGA and GEO datasets. Transl Androl Urol. 2024;13(12):2771-86.

79. Hou Y, Wang S, Zhang Y, Huang X, Zhang X, He F, et al. Proteomics Identifies LUC7L3 as a Prognostic Biomarker for Hepatocellular Carcinoma. Curr Issues Mol Biol. 2024;46(5):4004-20.
