# Supplementary figures and images for: Loss of DIAPH3 accelerates glioma genesis in mice
Source: Cell Death Dis. 2026 Mar 23;17(1):342. doi: 10.1038/s41419-026-08652-x (PMC13040077; doi:10.1038/s41419-026-08652-x)

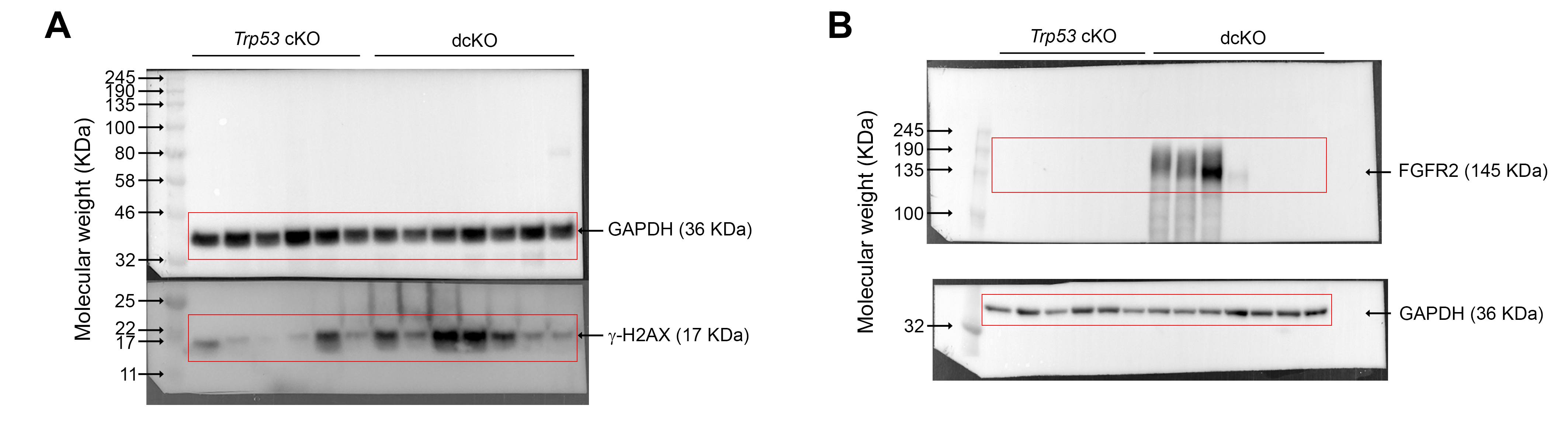

Supplement: Supplementary file 6 — Supplementary File 1 [file 41419_2026_8652_MOESM6_ESM.jpg]
